# Metagenomic data of bacterial community from different land uses at the river basin, Kelantan

**DOI:** 10.1016/j.dib.2020.106351

**Published:** 2020-09-28

**Authors:** Rennielyn Rupert, Grace Joy Chin Wei Lie, Daisy Vanitha John, Kogila Vani Annammala, Jaeyres Jani, Kenneth Francis Rodrigues

**Affiliations:** a Biotechnology Research Institute, Universiti Malaysia Sabah, Jalan UMS, Kota Kinabalu, Sabah 88400, Malaysia; b Department of Neuromicrobiology, National Institute of Mental Health and Neurosciences, Bangalore, India; c Centre for Environmental Sustainability and Water Security (IPASA), Universiti Teknologi Malaysia, Johor, Malaysia; d BorneoMedical and Health Research Center, Universiti Malaysia Sabah, Kota Kinabalu, Sabah 88400, Malaysia

**Keywords:** Metagenomics, Clustering analysis, Taxonomy tree, Land-uses, Kelantan river basin

## Abstract

The data provided in the article includes the sequence of bacterial 16S rRNA gene from a high conservation value forest, logged forest, rubber plantation and oil palm plantation collected at Kelantan river basin. The logged forest area was previously notified as a flooding region. The total gDNA of bacterial community was amplified via polymerase chain reaction at V3-V4 regions using a pair of specific universal primer. Amplicons were sequenced on Illumina HiSeq paired-end platform to generate 250 bp paired-end raw reads. Several bioinformatics tools such as FLASH, QIIME and UPARSE were used to process the reads generated for OTU analysis. Meanwhile, R&D software was used to construct the taxonomy tree for all samples. Raw data files are available at the Sequence Read Archive (SRA), NCBI and data information can be found at the BioProject and BioSample, NCBI. The data shows the comparison of bacterial community between the natural forest and different land uses.

## Specifications Table

**Table:** 

Subject	Biology
Specific subject area	Metagenomics, Bacteriology
Type of data	Table and figure
How data were acquired	The 16S rRNA metagenomic sequencing was conducted on Illumina HiSeq paired-end platform, and OTU clustering analysis was conducted using QIIME platform.
Data format	Raw and analysed data
Parameters for data collection	The gDNA of bacterial community in high conservation value forest, logged forest, rubber plantation and oil palm plantation at Kelantan river basin were identified using powersoil DNA Isolation kit, Phusion® High-Fidelity PCR Master Mix and TruSeq®DNA PCR Preparation Kit.
Description of data collection	The raw reads were trimmed and merged using FLASH software, and passed the quality control. Sequence with ≥97% similarity was categorized into similar OTUs. Representative sequence for each OTU was screened for species annotation using GreenGene Database based on RDP classifier algorithm to annotate taxonomic information. Taxonomic rank tree was constructed using R&D software.
Data source location	The soil samples were collected at various locations in river basin, Kelantan as followed: • High Conservation Forest (HCV): 05° 12.974′ N 102° 11.716′E • Logged forest (LF): 05° 13.228′ N 102° 11.592′ E • Rubber Plantation (RP): 05° 00.893′ N 102° 19.929′ E • Oil palm plantation (OP): 04° 56.197′ N 102° 24.472′ E
Data accessibility	The raw sequencing data is available at BioProject, BioSample and SRA, NCBI at https://www.ncbi.nlm.nih.gov/bioproject/?term=PRJNA448364 under the accession number of PRJNA448364 (BioProject).

## Value of the Data

• This data information provides the bacterial community of the primary forest and different land uses at the river basin, Kelantan.
• The data is applicable as a comparative study on the soil changes caused by different land use that are conducted in river basin, Kelantan.
• This data can be used to evaluate the soil conditions in the river basin based on the bacterial community that underlies in the soils.

## 1 Data Description

The data reported here are the sequence information and taxonomy assignment of bacterial community in four sampling sites with different soil types. Each of the sampling sites has six replicates of soil sample resulting to four sets of metadata. After sequencing, there was a total of 3 556 042 reads generated from the 24 samples, with a maximum 159 900 and a minimum 130 742 reads per sample. Among the reads, 2 869 264 reads were successfully processed into effective tags with 239 518 of unique tags and 216 of unclassified tags.

The processed tags were used for subsequent OTU analysis. Operational Taxonomic Units (OTU) is defined as a cluster of similar sequence read based on the taxonomic lineage that helps in analyzing species community in a sample [1]. The tags were clustered into OTUs by 97% DNA sequence similarity giving an average of 3, 271 OTUs per sample. Based on the OTU data, the top 10 dominant genus in high relative abundance were selected to construct a taxonomy tree. The construction of the tree helps in identifying the structure of bacterial community in different land uses in the Kelantan river basin.

## 2 Experimental Design, Materials and Methods

### 2.1 Sampling sites and collection

Soil samples were collected from four different locations, which are primary forest (high conservation value forest), logged forest (flooding area), rubber plantation and oil palm plantation. Soil was collected within a quadrant of 1 × 1 m^2^ with the depth approximately 20 cm from the soil surface. For each main site, six replicates were collected and placed into separate 50 ml of sterile tubes. Afterwards, the samples were stored in a cooler box and transported back to the laboratory.

### 2.2 DNA extraction, library preparation and sequencing

The total genomic DNA from the soil sample were extracted using powersoil DNA Isolation kit (MoBio Laboratories, USA) [2]. The quality of extracted gDNA was monitored on 2% agarose gels to check for its concentration and purity. For amplicon generation, bacterial 16S rRNA gene of selected regions V4-V5 were amplified using universal primers, 515F (GTGCCAGCMGCCGCGGTAA) and 926R (CCGTCAATTCMTT- TRAGTTT) [3]. Then, PCR reactions were carried out using Phusion® High-Fidelity PCR Master Mix (New England Biolabs). For library preparation, TruSeq®DNA PCR Preparation Kit (Illumina, USA) was used to generate the sequencing library. The quality of the library was assessed using Qubit@ 2.0 Fluorometer (Thermo Scientific) and Agilent Bioanalyzer 2100 system prior to sequencing. The library was ready to sequence on an Illumina Hiseq 2500 platform and 250 bp paired-ends reads were generated [4].

### 2.3 Data analysis

The raw reads were assigned to samples by matching them using their unique barcode and truncated them by trimming the barcode and primer sequence. The trimmed pair-end reads were merged using FLASH software [5]. High-quality clean tags were obtained by data filtering under specific filtering conditions according to QIIME quality-controlled process [6]. The clean tags were compared to a reference database (Gold Database) using UCHIME algorithm for chimera sequences detection [7]. Effective Tags were finally obtained after chimera removal. For OTU cluster and sequence annotation, the analysis was performed using Uparse software [8]. Sequences that possessed more than 97% similarity were grouped into the same OTUs. Each OTU's representative sequence was screened for species annotation using GreenGene Database based on RDP classifier algorithm to annotate taxonomic information [9]. Taxonomy tree for four main sampling sites were constructed based on the top 10 phyla in high relative abundance by independently R&D software. The study of phylogenetic relationships among the OTUs and differences of the dominant species in different groups were conducted by aligned multiple sequences using the MUSCLE software [10].

Fig 1 and Table 1

**Fig. 1:**
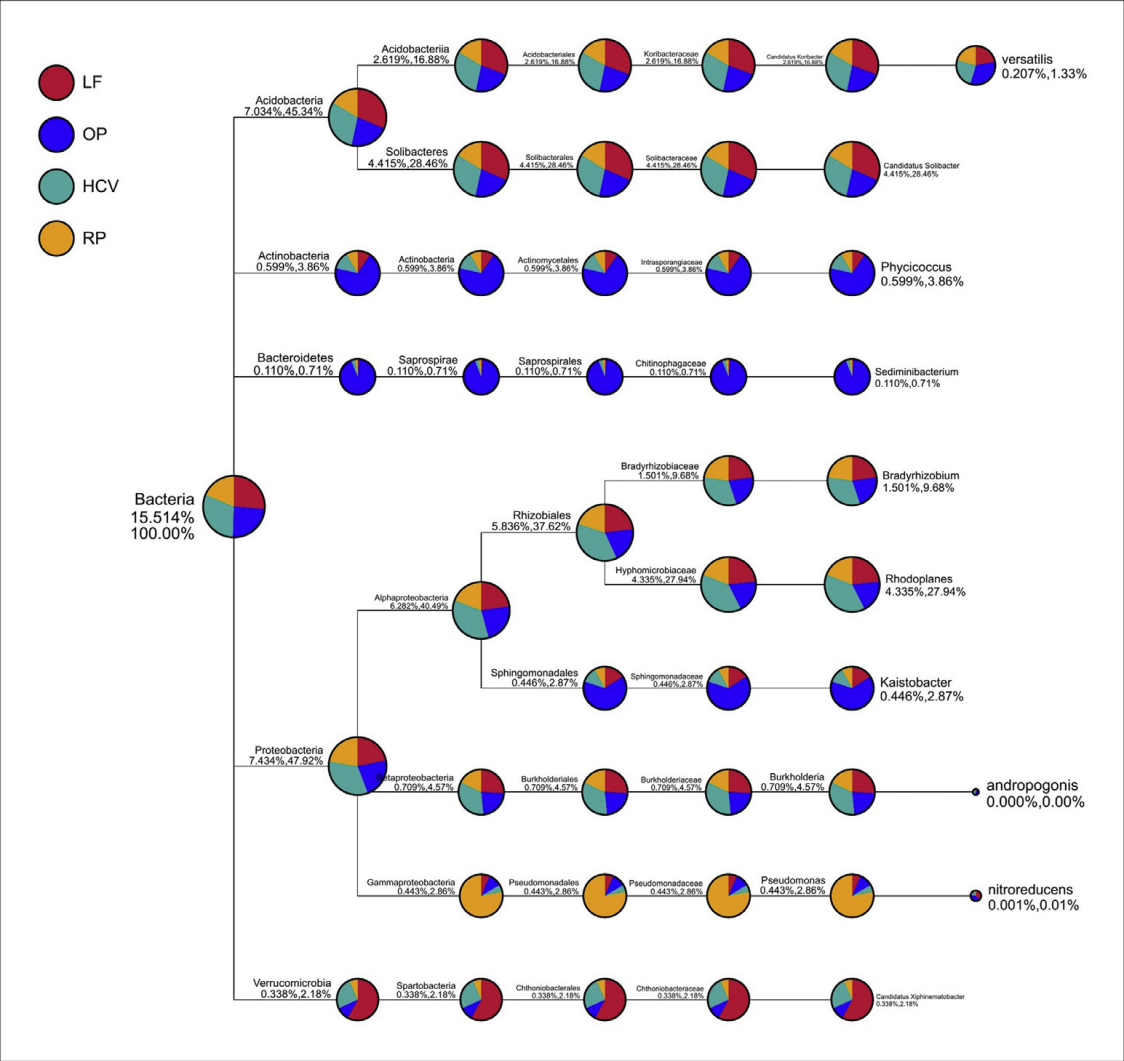
demonstrates the combined taxonomy trees in all samples. Sectors with different colours represent different sampling sites. Red color represents logged forest, blue represents oil palm plantation, turquoise represents high conservation forest, and orange represents rubber plantation. The size of the sector indicates the relative abundance. The first number below the taxonomic name represents the percentage in the whole taxon, while the second number represents the percentage in the selected taxon. Based on the taxonomic tree, the majority of phyla identified in the 24 samples were constituted of Proteobacteria (47.92%), Acidobacteria (45.34%), Actinobacteria (3.86%), Verrucomicrobia (2.18%) and Bacteroidetes (0.71%). (HCV = high conservation forest; LF = logged forest; RP = rubber plantation; OP = soil palm plantation).

**Table 1:** shows the summary of sequence information including the sample ID, Bioproject, Biosample, and SRA accession numbers assigned to the metadata.

Category	BioProject No.	BioSample No.	Sample ID	SRA No.
High conservation forest	PRJNA448364	SAMN08828866	HCV1	SRX3895719
			HCV2	SRX3895720
			HCV3	SRX3895721
			HCV4	SRX3895722
			HCV5	SRX3895715
			HCV6	SRX3895716
Logged forest		SAMN08828869	LF1.1	SRX3895717
			LF1.2	SRX3895718
			LF1.3	SRX3895713
			LF2.1	SRX3895714
			LF2.2	SRX3895732
			LF2.3	SRX3895731
Rubber plantation		SAMN08828884	RP1	SRX3895723
			RP2	SRX3895724
			RP3	SRX3895712
			RP4	SRX3895711
			RP5	SRX3895710
			RP6	SRX3895709
Oil palm plantation		SAMN08828882	OP1.1	SRX3895729
			OP1.2	SRX3895730
			OP2.1	SRX3895727
			OP2.2	SRX3895728
			OP3.1	SRX3895725
			OP3.2	SRX3895726

## Declaration of Competing Interest

The authors declare that they have no known competing financial interests or personal relationships which have, or could be perceived to have, influenced the work reported in this article.

## Appendix Supplementary Materials

Image, application 1
